# Integrated breast massage versus traditional breast massage for treatment of plugged milk duct in lactating women: a randomized controlled trial

**DOI:** 10.1186/s13006-022-00485-6

**Published:** 2022-06-02

**Authors:** Nutchanat Munsittikul, Supannee Tantaobharse, Pitiporn Siripattanapipong, Punnanee Wutthigate, Sopapan Ngerncham, Buranee Yangthara

**Affiliations:** 1 Pediatric Nursing Division, Nursing Department, Siriraj Hospital, Bangkok, Thailand; 2 Division of Neonatology, Department of Pediatrics, Faculty of Medicine Siriraj Hospital, Mahidol University, 2 Wanglang Road, Bangkoknoi, Bangkok, 10700 Thailand

**Keywords:** Integrated breast massage, Traditional breast massage, Treatment, Plugged milk ducts, Lactating women

## Abstract

**Background:**

Plugged milk duct during lactation is a common problem in breastfeeding. Traditional breast massage (TBM) has been performed in Thailand with reasonable outcomes, but several follow-up sessions are often required. A new massage technique, the integrated breast massage (IBM), was subsequently developed. This study aimed to compare resolution time, reduction in mass size, and pain score after breast massage between the IBM and TBM techniques.

**Methods:**

This randomized controlled trial was conducted at the Lactation Clinic of the Department of Pediatrics, Faculty of Medicine Siriraj Hospital, Mahidol University, Bangkok, Thailand during February 2019-July 2020. Women presenting with acute plugged milk duct were enrolled and randomly allocated to the IBM or TBM/control groups. Mass size in square centimeters (cm^2^) was calculated by multiplying the perpendicular axes of the mass. Pain score was self-scored by participants using a numerical rating scale. Median time (95% confidence interval [CI]) to resolution of plugged milk duct was derived using Kaplan–Meier survival analysis. Intention-to-treat analysis was performed.

**Results:**

Eighty-four women (42 per group) were included. All enrolled study participants completed the study and were included in the final analysis. Twenty-six (61.9%) and 25 (59.5%) participants from IBM and TBM, respectively, had mass diameter > 5 cm. The median (interquartile range [IQR]) mass size was 30 (20–48) and 20 (12–14) cm^2^ in IBM and TBM (*p* = 0.05), respectively. The median (95% CI) time to resolution of plugged duct was 0 (not available) and 1 (0.47–1.53) day in IBM and TBM, respectively (*p* < 0.01). After the first breast massage, the median (IQR) size of mass reduction was 30 (20–48) and 10 (10–26) cm^2^ in IBM and TBM, respectively (*p* = 0.01). The median (IQR) reduction in pain score was 8 (7–8) and 6 (4–7) in IBM and TBM, respectively (*p* = 0.01). No participants developed skin bruising or hematoma after breast massage.

**Conclusions:**

The IBM technique resolved plugged milk duct significantly faster, with significantly less pain, and with significantly greater reduction in mass size after the first massage compared to TBM.

**Trial registration:**

Retrospectively registered in the Thai Clinical Trials Registry on 25 September 2019 (TCTR20190925001).

## Background

Breastfeeding problems that cause maternal pain, such as breast engorgement, plugged milk duct, and mastitis, are important causes of premature breastfeeding cessation [1, 2]. Plugged milk duct is one of the five most common breastfeeding problems encountered at the lactation clinic, Siriraj Hospital, Bangkok, Thailand at 45.9%, unpublished data collected during January to December 2016, included 135 lactating women with breast problems. Plugged milk duct causes local milk stasis, which leads to the development of painful breast masses [3]. Plugged milk duct is one of the risk factors for mastitis, and it should be corrected within 48 h of breast mass onset [4].

Therapeutic breast massage in lactation (TBML), which consists of gentle breast massage motions toward the axillary area, stimulates lymphatic and blood circulation, increases breast milk production, and facilitates the resolution of plugged milk duct [2]. TBML was first introduced at the 5^th^ National Breastfeeding Conference in 2016 in Thailand. Since then, traditional breast massage (TBM) was developed and adopted by several breastfeeding clinics in Thailand, including ours. However, we found that up to four days of daily TBM was required to resolve plugged milk duct in some patients. To hasten the resolution of plugged milk duct in lactating mothers, our team developed a new technique called integrated breast massage (IBM). This technique combines the sequential performance of several different massage patterns introduced by Ma. Ines Av. Fernandez to improve lymphatic and blood circulation [5]. We added nipple rolling to promote milk duct dilatation and in the final step, manual breast mass immobilization and gentle pressing to effectuate drainage of the mass of accumulated milk. The technique is described in more detail in the Methods section below.

The primary aim of this study was to compare the time to mass resolution between IBM and TBM in lactating women with plugged milk duct. The secondary objectives were to compare pre- and post-massage mass size and pain scores between the IBM and TBM groups.

## Methods

### Study design, setting, and participants

This single-center, single-blinded, stratified randomized controlled trial was conducted at the Siriraj Lactation Clinic of the Department of Pediatrics, Faculty of Medicine Siriraj Hospital, Mahidol University, Bangkok, Thailand during February 2019 to July 2020. Siriraj Hospital is Thailand’s largest national tertiary referral center with approximately 8,000 deliveries per year. Our center’s lactation clinic, the Siriraj Lactation Clinic, attends to approximately 2,000 cases of breastfeeding associated problems per year.

Eligible participants were lactating women who presented at the Siriraj Lactation Clinic with plugged milk duct that had developed within 72 h of presentation. Women who had been previously treated with antibiotics, who had received breast massage elsewhere, who had surgery on the ipsilateral breast, or who had mastitis or breast abscess were excluded. Study participants were stratified according to the widest part of the mass, measured by a caliper, (the widest part of the largest mass if several masses were present) to more than or equal to 5 cm or less than 5 cm. This stratification was based on findings from the study of lactational breast abscess that the abscess size of more than 5 cm was a risk factor for failure of treatment by needle aspiration [6]. This study complied with all of the principles set forth in the 1964 Declaration of Helsinki and all of its subsequent amendments, and written informed consent was obtained from all study participants. The protocol for this study protocol was approved by the Siriraj Institutional Review Board (SIRB) (COA no. SI065/2019), and was retrospectively registered in the Thai Clinical Trials Registry, which is one of the primary registries of the WHO Registry Network. The study registration number is TCTR20190925001.

### Breast massage procedure

The affected breast was covered with a wet warm towel (except for the nipple area) for 10 min, and breastmilk was removed by electrical pump (Symphony®; Medela AG, Baar, Switzerland) or by breastfeeding the participant’s baby for 30 min. Two registered nurses were committed to a specific breast massage technique throughout the entire study, NM for IBM and ST for TBM. Before starting the massage, the study participant was in the supine position with the head elevated at a 45^o^ angle. The nurse performing the massage was position beside the patient’s head. The massage pressure would reach skin and superficial fascia levels and the underlying muscles or bone should never be palpated [7]. The pressure was generated from the massager’s hands, arms, and shoulders, not from the massager’s body weight by leaning toward the participants during the massage. The massagers took great care to adjust the pressure according to the participant’s comfort, which were continuously assessed by the massagers.

A 30-min session of assigned breast massage was given by the assigned nurse in a private room away from other nurses working in the clinic. To blind the outcome assessor, both study massage nurses were in the room with the participant at the same time, so the assessor did not know which type of massage the patient received during the session. Participants could not be blinded to the massage technique they received.

### Traditional breast massage (TBM) [2]

Step 1: The massager places her hands on the affected breast and gently massages around the breast for 5 rounds to relax the participant.

Step 2: Starting from the base of the breast, use the middle 3 fingers to lightly press and massage in a circular motion. Move around to cover the entire breast from its base ascending toward the nipple for five minutes.

Step 3: Manually express breast milk until slow flow is achieved, followed by a repeat of steps 1–3 until the end of the 30-min session.

### Integrated breast massage (IBM) technique (Fig. 1)

**Fig. 1:**
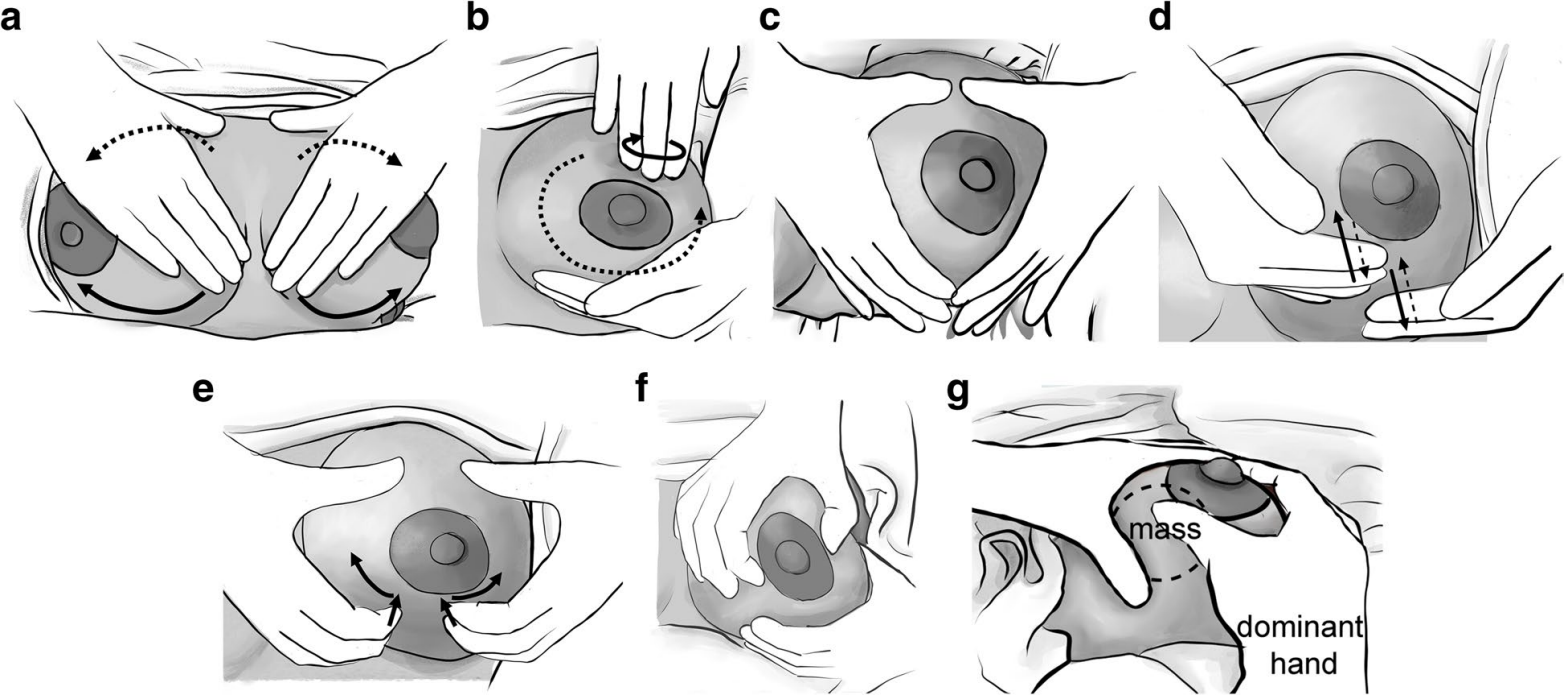
Pictorial images of the integrated breast massage technique with the massage therapist standing behind the patient’s head. **A** *Butterfly stroke*: Apply continuous pressure to the affected breast while moving along the base of the breast from the medial side to the axillary area. Then repeat the same motion at the upper half of the breast. **B** *Fingertip circle*: With the fingers of one hand, lightly press and massage the breast in a circular motion while moving around the areola. **C, D, E** Diamond stroke. **C**: The breast is positioned between the fingers and thumbs of both hands (resembling a diamond shape), followed by both hands moving toward each other and toward the areola. **D**: Alternating between hands, press the breast with the palmar side of the 2^nd^ to 5^th^ fingers, and then release (resembling patting on the breast). **E**: With the tips of the 2^nd^ to 5^th^ fingers, press into the breast gently, then move both hands away from each other with an action that is similar to scratching. Repeat the same motions around the breast toward the axillary area. **F** *Promotion of milk duct dilatation*: Gently squeeze and roll the areolar area between the thumb and forefinger. **G** *Augmentation of milk drainage from areas with plugged milk ducts*: Gently fix and squeeze the breast mass with the non-dominant hand while the dominant hand manually expresses breast milk by gently compressing the areola and nipple between the thumb and index finger

Step 1 to 3 of the IBM technique was described by Ma. Ines Av. Fernandez, an executive director of Arugaan Foundation, Philippines, in her presentation at the 4^th^ National Breastfeeding Conference in Thailand in 2013 [5]. From step 1 to step 3, took approximately 5 min.

Step 1: *Butterfly stroke* – Apply continuous pressure to the affected breast while moving along the base of the breast from the medial side to the axillary area. Then repeat the same motion at the upper half of the breast.

Step 2: *Fingertip circle* – With the fingers of one hand, lightly press and massage the breast in a circular motion while moving around the areola and supporting under the breast with the other hand.

Step 3: *Diamond stroke* – The breast is positioned between the fingers and thumbs of both hands (resembling a diamond shape), followed by both hands moving toward each other and toward the areola. Alternating between hands, press the breast with the palmar side of the 2^nd^ to 5^th^ fingers, and then release (resembling patting on the breast). With the tips of the 2^nd^ to 5^th^ fingers, press into the breast gently, then move both hands away from each other with an action that is similar to scratching. Repeat the same motions around the breast toward the axillary area.

Step 4: *Promotion of milk duct dilatation and augmentation of milk drainage from areas with plugged milk ducts* – Gently squeeze and roll the areolar area between the thumb and forefinger. Gently fix and squeeze the breast mass with the non-dominant hand while the dominant hand manually expresses breast milk by gently compressing the areola and nipple between the thumb and index finger.

### Follow-up schedule

Participants underwent pain assessment, mass measurement, and one session of breast massage every time they visited the clinic. After the first visit, participants were followed-up daily until the breast mass was no longer palpable, and then they were interviewed by telephone at 3–5 days after the last visit. Study women were advised to return to the clinic if a breast mass reemerged or if experiencing breast pain. All participants were instructed to regularly express breast milk by hand, breast pump, or breastfeeding every three hours (total of 8 times per day), and to maintain a daily record of milk expression time and frequency. Ibuprofen and/or acetaminophen was prescribed for pain control.

### Outcome measurements and operational definitions

At recruitment, a single assigned nurse who was not involved with the trial and who was blinded to the intervention measured the breast mass with a caliper at its widest x and y axes. Mass size in square centimeters (cm^2^) was calculated by multiplying the x-axis distance by the y-axis distance. Because changes in mass diameters in x and y axes after the interventions may not be proportional, the multiplication product of both diameters should better represent changes in mass size. Before massage, participants were asked to rate their breast pain according to a 0-to-10 numerical rating scale (NRS), with zero indicating no pain, and a 10 indicating the worst possible pain. At the end of each breast massage session, participants were asked to once again rate their level of breast pain. The same assigned nurse then remeasured the size of the breast mass. At each visit, the participants were examined for clinical signs of mastitis or breast abscess, inquired for analgesics use, and compliance in breast milk drainage.

Plugged milk duct was diagnosed clinically by the development of acute painful breast masses (localized milk stasis), which could be accompanied by mild redness of the covering skin and low-grade fever (≤ 38.4 °C) [3]. Breast abscess was distinguished from plugged milk ducts by the presence of higher-grade fever (> 38.4 °C), fluctuation of the mass upon palpation, and severe pain upon gentle palpation [3]. If a clinical diagnosis of breast abscess was uncertain, diagnostic breast ultrasound would be performed to determine the diagnosis. Complete resolution of plugged milk duct was defined as no recurrent breast mass in the same area within the follow-up period of the study. Recurrence that developed after the end of the study follow-up period was considered a new episode of plugged milk duct.

### Sample size calculation and statistical analysis

The sample size was calculated based on a survival time design using data from our pilot study. The preliminary results showed a median time to resolution of four and two days after treatment with TBM and IBM, respectively. The specified accrual time and additional follow-up time after the accrual interval was 60 days and five days, respectively. Forty-two participants were required in each arm to obtain detection power of 80%, type I error of 0.05, and to compensate for an estimated possible 20% drop-out rate [8].

Block of 4 randomization was performed by a statistician using the nQuery software program (Statistical Solutions Ltd, Cork, Ireland). Sealed opaque envelopes were used to conceal the study arm assignment of each enrolled study patient. Participants were to be withdrawn from the trial if mastitis or breast abscess developed at any point during the study. As a rescue treatment, participants could be switched to the other arm at any time if the reduction in mass size was less than 20% after receiving the assigned breast massage technique at that visit.

Descriptive statistics were used to summarize the demographic and clinical characteristics of study women. Categorical data were presented as number and percentage and were compared using chi-square test or Fisher’s exact test, depending on the size of the sample. Normally and non-normally distributed continuous data were demonstrated as mean plus/minus standard deviation (SD) and median and interquartile range (IQR), respectively. These continuous data were compared using Student’s *t*-test and Mann–Whitney U test, respectively. After Kaplan–Meier survival curve analysis, differences in plugged milk duct resolution time were analyzed by log rank test. All analyses were performed in an intention-to-treat manner. SPSS software version 18.0 (SPSS, Inc., Chicago, IL, USA) was used for statistical analysis, and Microsoft Excel version 2019 (Microsoft Corporation, Redmond, WA, USA) was used for graphical presentation. A *p*—value less than 0.05 was considered statistically significant for all tests.

## Results

**Fig. 2:**
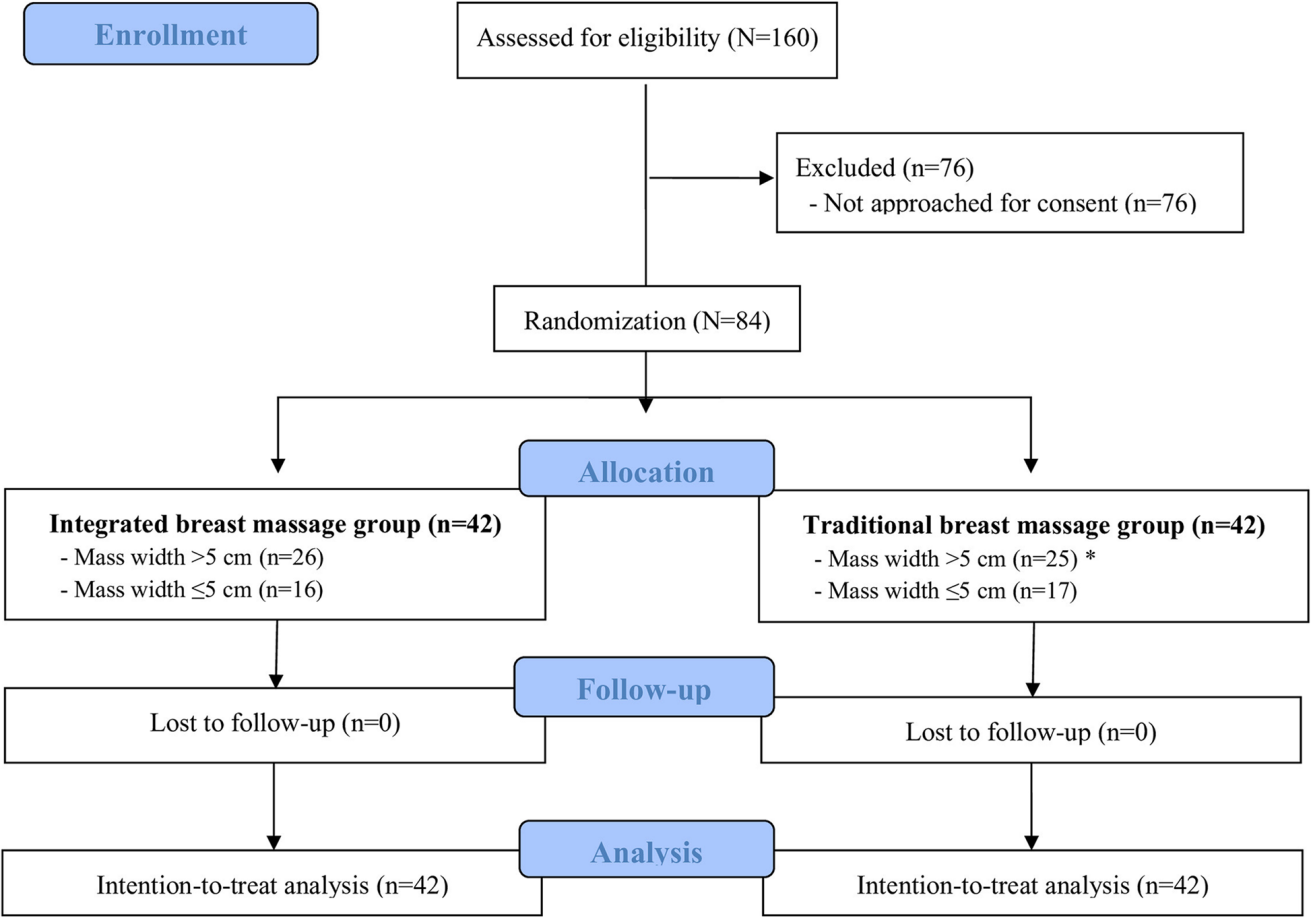
Diagram demonstrating the flow of participants in this study. *Two cases in the traditional breast massage group received integrated breast massage on the second visit due to their experiencing a < 20% reduction in mass size after receiving traditional breast massage

During the February 2019 to July 2020 study period, 340 women with plugged milk duct were eligible to join the study; however, the study was paused for seven months due to the maternity leave of one nurse and study leave of the other nurse, both of whom perform breast massage. Of the 160 women who were assessed for eligibility during the recruitment period, 84 women were enrolled in this study. Of the 84 study women, 42 were randomized to the IBM group, and the other 42 were randomized to the TBM group, which was the control group (Fig. 2). No study women were lost to follow-up.

Demographic and clinical characteristics of all participants and compared between the TBM and IBM groups are shown in Table 1. The median age of participants was 34 years (IQR: 29–36), and the median age was comparable between groups. Eighty percent of participants breastfed their infants. Most participants also expressed milk by electric breast pump. Onset of plugged milk duct ranged from 25–72 h prior to presentation at our clinic in 96.4% of participants. Most participants presented with only one breast mass (71/84, 84.5%) with a median size of 25 cm^2^ (IQR: 16–42). Twenty-six (61.9%) and 25 (59.5%) participants from IBM and TBM, respectively, had breast mass diameter of more than 5 cm. Women in the IBM group had a significantly larger median breast mass size [30.0 cm^2^ (IQR: 20.0–48.25) *vs.* 20.0 cm^2^ (IQR: 12.0–42.0), *p* = 0.045], and a significantly higher median NRS pain score before massage [8 (IQR: 7.8–8) *vs.* 7 (IQR: 5–8), *p* < 0.01] compared to women in the TBM group.

**Table 1:** Demographic and clinical characteristics of all participants and compared between the traditional and integrated breast massage groups

Characteristics	Total (*N* = 84)	Traditional Breast Massage (*n* = 42)	Integrated Breast Massage (*n* = 42)	*p*-value
Age (years), median (IQR)	34.0 (29.0–36.0)	33.0 (29.0–36.0)	34.0 (31.0–36.0)	0.42
Milk expression method before onset of plugged duct, n (%)				
Breastfeeding	67 (79.8%)	37 (88.1%)	30 (71.4%)	0.06
Hand expression	11 (13.1%)	2 (4.8%)	9 (21.4%)	***0.02***
Breast pump	74 (88.1%)	36 (85.7%)	38 (90.5%)	0.50
Time of onset prior to presentation at our clinic, n (%)				0.29
≤ 24 h	3 (3.6%)	3 (7.1%)	0 (0.0%)	
25–48 h	48 (57.1%)	24 (57.1%)	24 (57.1%)	
49–72 h	33 (39.3%)	15 (35.7%)	18 (42.9%)	
More than 1 mass, n (%)	13 (15.5%)	4 (9.5%)	9 (21.4%)	0.35
Size of the largest mass (cm^2^), median (IQR)	25.0 (16.0–42.0)	20.0 (12.0–42.0)	30.0 (20.0–48.25)	***0.045***
NRS pain score before massage, median (IQR)	8 (7–8)	7 (5–8)	8 (7.8–8)	** < *0.01***
Cracked nipples and/or nipple blebs, n (%)	11 (13.1%)	5 (11.9%)	6 (14.3%)	1.00

A *p*-value < 0.05 indicates statistical significance

Abbreviations: *IQR* interquartile range, *NRS* numerical rating scale

**Fig. 3:**
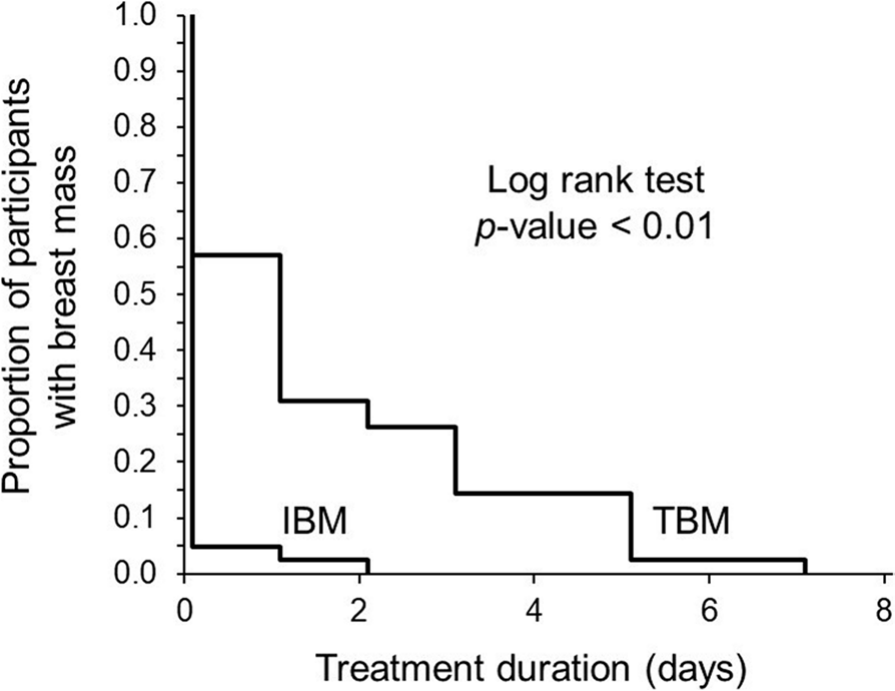
Kaplan–Meier survival curve analysis comparing the duration of plugged milk duct treatment between integrated breast massage (IBM) and traditional breast massage (TBM)

**Fig. 4:**
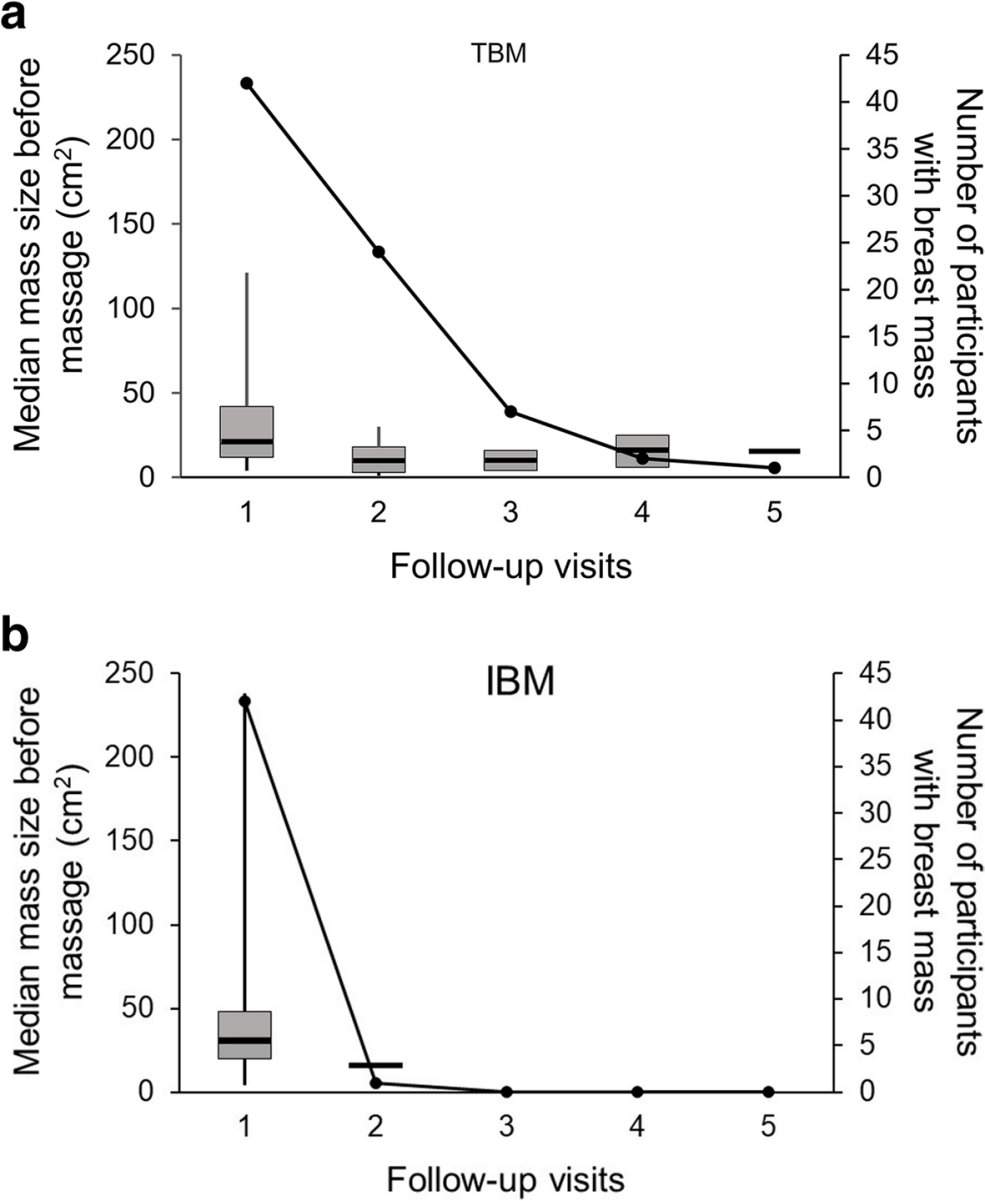
Change in median mass size (box plot) and number of participants with remaining breast mass (line plot) at each follow-up visit. **A** TBM, traditional breast massage and **B** IBM, integrated breast massage

The median time to resolution of plugged milk duct was 1 day (95% CI 0.47–1.53) for TBM women, and 0 days (95% CI 0–0) for IBM women (*p* < 0.01) (Fig. 3). Breast mass was completely resolved after the first massage session in 24/42 (57.1%) and 41/42 (97.6%) participants in the TBM and IBM groups, respectively (*p* < 0.01). All participants had complete resolution of breast mass after the 5^th^ and 2^nd^ visits in the TBM and IBM groups, respectively (Fig. 4).

After the first breast massage session, the median reduction in mass size was significantly greater in the IBM group than in the TBM group (30 cm^2^ [IQR: 20–48] or 100% (IQR: 100–100) of the original size *vs.* 10 cm^2^ [IQR: 10–26] or 89.3% [IQR: 65.9–100.0] of the original size, respectively; *p* = 0.01). The median reduction in NRS pain score after the first breast massage was also significantly greater in the IBM group (8 [IQR: 7–8] or 100% [IQR: 100–100] of the original score *vs*. 6 [IQR: 4–7] or 95% [IQR: 75–100], respectively; *p* = 0.01). After the first breast massage, prescribed pain medication was taken by 3/42 (7.1%) and 1/42 (2.4%) participants in the IBM and TBM groups, respectively (*p* = 0.62). No study participants developed skin bruising or hematomas after massage. Two participants switched from the TBM group to the IBM group during the 2^nd^ visit due to those women experiencing a less than 20% reduction in mass size after undergoing TBM at the first visit (Fig. 2).

No participants had recurrent breast mass, developed mastitis, developed breast abscess or required treatment with antibiotics during the study follow-up period. The frequency of daily breast milk expression in the TBM group during the follow-up period was eight times/day (IQR: 6–8). The one participant in the IBM group who required a follow-up visit expressed breast milk six times per day. Because only one participant in the IBM group required a follow-up visit, a comparison of milk expression frequency between the IBM group and the TBM group was not performed.

## Discussion

Compared to the TBM technique, the IBM technique yielded a significantly shorter median time to resolution of plugged milk duct, and a significantly higher percentage of study women who achieved complete resolution after one massage session. The IBM technique also resulted in a significantly greater reduction in median mass size and median NRS pain score after massage compared to the TBM group. Neither technique resulted in hematoma or bruising of the breast. No participants had recurrent plugged milk duct, developed mastitis, or developed breast abscess during the study follow-up period.

Our results suggested the superiority of the IBM technique over the TBM technique relative to time to resolution of plugged milk duct. This may be explained by two possible factors. First, the addition of the last step in which pressure is applied directly to the breast mass resulted in improve drainage of milk. Second, enhancement of steps designed to improve lymphatic and blood circulation to promote dilatation of the milk duct. Witt, et al. [2] found that a session of TBML (the technique from which TBM was derived) at a median massage duration of 30 min could completely resolve plugged milk duct in 16/28 (57.1%) participants, which is similar to the results in our TBM group 18/42 (42.9%) after the first visit. This similarity between study findings indicates that even though TBM is a modified version of TBML, the shared concept of improving lymphatic and blood circulation resulted in comparable favorable effects. Application of pressure directly to the breast mass in another technique that is called 6-step recanalization manual therapy (SSRMT) was also shown to yield similar outcomes [9]. Ninety-one percent of subjects who received SSRMT experienced complete resolution of plugged milk duct after just one massage session. The SSRMT technique aims to clear a clogged milk duct via several steps of nipple and areolar manipulation before kneading and pushing the breast in the last step in an attempt to express the captive milk [9]. Similar to the SSRMT technique, our IBM technique includes breast preparation steps dedicated to enhancing milk flow before the application of direct mass pressure. However, instead of focusing solely on nipple and areolar manipulation as in SSRMT, our preparation steps aim to improve breast lymphatic and blood circulation using butterfly, finger circle, and diamond strokes, followed by an attempt to enhance milk duct dilatation via areolar massage. Similar to our technique, Russian breast massage also includes preparation steps to enhance lymphatic and blood circulation before applying pressure to the edge of the breast mass [10]. No or inadequate breast preparation could result in intolerable pain during the application of pressure to the mass [11]. The finding that participants in our study and in SSRMT studies required no pain medications during the procedure may suggest the importance of breast preparation before application of direct pressure to the mass.

Massage techniques, such as ours, that require the direct application of pressure to the breast mass might be more suitable as a clinic-based therapy because expertise is required to avoid unbearable pain and tissue injury. However, TBM and TBML have the potential of being at-home self-massage techniques. In the present study, over half of patients in the TBM group experienced complete resolution after just one session. However, a breast mass with a diameter of greater than 5 cm might respond poorly to TBM, as shown in our study and as evidenced by two participants who had to be switched from the TBM group to the IBM group.

The shorter resolution time associated with the IBM technique might confer some important benefits, including faster restoration of breastmilk volume and higher probability of regaining infant latch on. However, after resolution of a plugged milk duct, an infant might continue to refuse feeding from the affected breast because milk stasis led to reduced lactose concentration, and increased sodium content for up to one week [12, 13]. Increased reduction in breast pain after breast massage should also contribute to the continuation of breastfeeding since breast pain is a major cause of early weaning [14].

Several studies in different breast massage techniques have been conducted, including Oketani massage [15], oxytocin massage [16], Gua-Sha [17], and acupressure [18]. All of those studies shared the similar objective of enhancing milk flow via distinct underlying principles. Further studies are needed to determine which of the breast preparation techniques is most effective, and which technique is least painful during the last pressure step. Further study is also needed to compare the effectiveness of in-clinic TBM or TBML with self-massage TBM or TBML as a treatment for plugged milk duct since uncertainties regarding the persistence of the COVID-19 pandemic may require that breast massage be self-performed at home after online training. However, reported limitations of this type of telemedical training include technical difficulty, small video screen that impairs the viewing of essential content, and harder to read non-verbal cues [19].

### Strengths and limitations

The main strength of this study is that it is the first randomized controlled trial to compare time to resolution of plugged milk duct between two breast massage techniques. Moreover, the study interventions were uniform throughout the entire study period because only two nurses with expertise in each technique were responsible for all massage sessions, and both nurses were in attendance during each massage session in both groups. The study was carefully randomized and designed to ensure that nurses who measured the outcome were blinded to the treatment arms.

The limitations of this study include its single-center design, the fact that no formal pain assessment was performed and recorded during the massage procedure, and that longer-term outcomes and complications, such as breastfeeding maintenance, and incidence of recurrent plugged milk duct, were not assessed. The fact that this study depended on individual expertise of only two nurses while in real-life practice, many more nurses were involved in breast massage for treatment of plugged milk duct, could be a study limitation as well as strength. Therefore, further studies are required to demonstrate reproducibility of IBM effectiveness in a real-world situation. The addition of a control group that does not receive breast massage, cool instead of warm compresses, and home massage could be considered in a future study. The control group with no breast massage is particularly important to confirm that the breast massage does not cause iatrogenic complications or increase pain.

## Conclusions

The IBM technique resolved plugged milk duct significantly faster, with significantly less pain, and with significantly greater reduction in mass size after the first massage compared to TBM. No study women had recurrent breast mass, developed mastitis, or developed breast abscess during the study follow-up period. Moreover, no study participants developed skin hematoma or bruising of the breast after massage. Therefore, the IBM technique should be considered a safe and effective treatment for resolving plugged milk duct in lactating women.

## Data Availability

The datasets used and/or analyzed during the current study are available from the corresponding author on reasonable request.

## Abbreviations

°C: Degrees Celsius
cm^2^: Square centimeters
COA: Certificate of approval
COVID-19: Coronavirus disease 2019
IBM: Integrated breast massage
IQR: Interquartile range
NRS: Numerical rating scale
SSRMT: Six-step recanalization manual therapy
TBM: Traditional breast massage
TBML: Therapeutic breast massage in lactation
TCTR: Thai Clinical Trials Registry
*vs.*: Versus
WHO: World Health Organization
